# Eigenstress model for electrochemistry of solid surfaces

**DOI:** 10.1038/srep26897

**Published:** 2016-06-03

**Authors:** Hongxin Ma, Xilin Xiong, Panpan Gao, Xi Li, Yu Yan, Alex A. Volinsky, Yanjing Su

**Affiliations:** 1 Corrosion and Protection Center, Key Laboratory for Environmental Fracture (MOE), University of Science and Technology Beijing, Beijing 100083, China; 2 Department of Mechanical Engineering, University of South Florida, Tampa, Florida 33620, USA

## Abstract

Thermodynamic analysis and molecular dynamics simulations were conducted to systematically study the size-dependent electrochemical response of solids. By combining the generalized Young-Laplace equation with the popular Butler-Volmer formulation, the direct influence of surface stress on solid film electrochemical reactions was isolated. A series of thermodynamic formulas were developed to describe the size-dependent electrochemical properties of the solid surface. These formulas include intrinsic surface elastic parameters, such as surface eigenstress and surface elastic modulus. Metallic films of Au, Pt, Ni, Cu and Fe were studied as examples. The anodic current density of the metal film increased, while the equilibrium potential decreased with increasing solid film thickness.

Surface stress/energy of solids plays a key role in the thermodynamics of solid surfaces, offering the description of macroscopic phenomena, including electrochemical and chemical responses. While the surface-to-volume ratio of nanomaterial is much larger than that of bulk material, this role becomes even more significant in nanomaterials. However, classical electrochemical theory does not completely account for the surface stress/energy of a solid.

Surface stress/energy of solids has been studied over half a century by many researchers^1^,^2^,^3^. When surfaces are created by cutting a crystal along a crystallographic plane, fresh surfaces without relaxation have a much higher surface energy. Atomic simulations^4^,^5^ and experiments^6^ have verified that energy minimization of the separated free-standing crystals occurs unavoidably to reduce the surface and the total energy. This relaxation rearranged atoms positions locally to lower the total energy, but may lead to a change in the lattice spacing of a nanometer-sized material, i.e. induce initial strain^7^. Surface relaxation was separated into the surface-normal relaxation and the surface-parallel relaxation^8^, which induces deformation parallel to the surface. After relaxation, a tensile (or compressive) surface eigenstress causes a compressive (or tensile) initial strain in the core of the nanomaterial with respect to its bulk lattice. Due to the initial deformation, both the surface energy density and surface stress are size-dependent. Weissmüller *et al*.^8^ proved that the surface-parallel and surface-normal deformations are both state variables of the surface free energy.

Dingreville *et al*.^9^,^10^ systematically studied the relationship between the surface energy, surface stress and surface elastic constants within the scheme of continuum mechanics theory. They incorporated the surface free energy into the continuum theory of mechanics demonstrated that the overall elastic behavior of the structural elements (particles, wires, and films) are size-dependent^9^. Although such size-dependency is negligible for conventional structural elements, it becomes significant when at least one of the dimensions of the element shrinks to nanometers. Dingreville *et al*.^11^ also analyzed the problem of the interfacial excess energy, excess stress and excess strain of planar interfaces. Their analysis revealed that the surface stress and the surface strain are intrinsic material properties. The in-plane interfacial stiffness tensor, the out-of-plane interfacial compliance tensor, and the coupling tensor, which accounts for the Poisson’s effect of the interface, are all independent of the solid geometrical size, and fully describe the elastic behavior of a coherent interface upon deformation.

Interactions between mechanical and electrochemical effects have an influence on the electrode reactions on metal surfaces^12^. Weissmüller *et al*.^8^ suggested that in terms of electrochemical experiments under pressure, the electrode potential is pressure-dependent. In some cases, mechanical and electrochemical effects are coupled with each other leading to modified electrochemical reaction rates or corrosion rates of the solid with respect to its stress-free state^13^. From the above discussion, it is reasonable to expect that the size-dependent surface energy/stress of the nanomaterial will lead to size-dependent electrochemical and corrosion properties. Actually, numerical and experimental results show that the electrochemical reaction rate of nanograined materials is size-dependent. As examples, the corrosion resistance properties of 304 stainless steel in NaCl solution^14^,^15^,^16^,^17^ and iron in alkaline solutions^18^ were improved when the material grain size was at the nanometer scale. Despite the same chemical composition, the breakdown potential of the sputtered nanocrystalline (grain size approximately 25 nm) 304 type stainless steel film is found to be approximately 850 mV higher than that of the conventional material.^16^ The high corrosion resistance of the sputtered film is attributed to the smaller grain size of the film. The similar results were reported by Youssef^19^, the estimated corrosion rate of nanocrystalline zinc (56 nm) with random orientation was found to be about 60% lower than that of electrogalvanized steel, 90 and 229 μA/cm^2^, respectively. In the work of Mishra^20^, the electrochemical and corrosion behavior of nanocrystalline nickel of different grain sizes (8–28 nm) in 1 mol/L H_2_SO_4_ electrolyte was compared with that of bulk Ni. The breakdown potential for fine grain sized nanocrystalline nickel was higher than that of coarse-grained polycrystalline nickel. There was a systematically increase in breakdown potential from 1110 mV to 1540 mV (silver-silver chloride reference electrode) with decreasing the grain size to 8 nm. The corrosion rate of freshly exposed nanocrystalline Ni was lower compared to that of bulk Ni, indicating a higher hindrance to anodic dissolution from the nanocrystalline Ni surfaces. On the other hand, an opposite trend was observed experimentally. The corrosion rate of Ti alloy in H_2_SO_4_ and HCl solutions was inversely proportional to the square root of the grain size^21^. The corrosion resistance of the Cu_90_Ni_10_ alloy in neutral Cl^−^-containing solution was reduced when the grain size was at the nanometer scale^22^. Thus, it is necessary to understand and predict the size-dependent electrochemical properties of nanomaterials, which is the subject of the present study.

Electrode reaction rates are commonly modeled using the Butler-Volmer equation^23^,^24^,^25^,^26^. The classical form of the Butler-Volmer equation does not incorporate the effect of the surface stress on the reaction rates. Gutman^12^ systematically studied the effect of the applied stress on the electrochemical response of bulk materials. In this paper, a stress-dependent chemical potential, obtained using the Gibbs-Duhem equation^27^, was used to extend the classical Butler-Volmer equation. The generalized Young-Laplace equation^28^ was used to describe the mechanical force balance between the surface and the underlying bulk material. By combining the Young-Laplace equation with the Gibbs-Duhem^27^ and the Butler-Volmer^23^,^24^,^25^,^26^ equations, the dependence of the electrochemical potential on the intrinsic surface elastic parameters, such as the surface eigenstress and surface elastic modulus, was obtained. Intrinsic surface elastic parameters were incorporated to reveal the physical origin of the size-dependent electrochemical corrosion properties, such as the current density of the electrode reactions, along with the equilibrium potentials of solid films.

## Results

### Intrinsic surface elastic parameters and thickness

**Figure 1:**
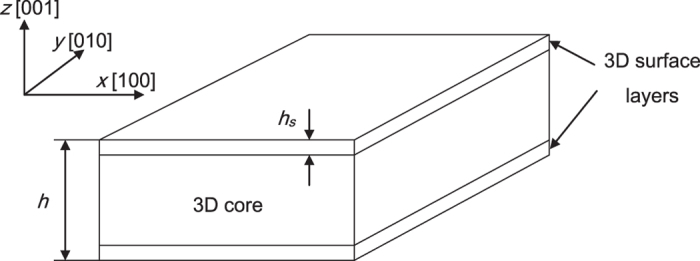
Model of a solid film with (001) surface separated into two 3D surface layers with the thickness *h*_s_ coherently adhered to the 3D core with the thickness *h* – 2*h*_s_.

A solid film with the (001) surface is modeled as a composite consisting of two 3D surface layers coherently bonded to a 3D core, as shown in Fig. 1. The core and the surface layers are assumed to be mechanically isotropic, linearly elastic, and the surface and core stresses are homogeneous. The orthogonal coordinates *x*, *y*, and *z* were set along the [100], [010], and [001] lattice directions, respectively.

Molecular dynamics (MD) simulations were conducted to extract the 2D intrinsic surface elastic parameters of 2D surface eigenstress, 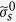, 2D surface biaxial Young’s modulus, 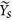, and bulk biaxial Young’s modulus *Y*_*c*_ of Pt, Au, Ni, Cu and Fe. The 3D intrinsic surface elastic parameters of 3D surface eigenstress, 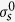, and 3D surface biaxial Young’s modulus, *Y*_*s*_, can be got by 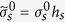 and 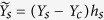, respectively. The intrinsic surface elastic parameters of the (001) surface were summarized in Table 1, with the assumption of the surface layer thickness *h*_s_ = 1 nm.

The 3D surface eigenstress and 3D surface biaxial Young’s modulus can be determined by above formulas, if one has known the thickness of the surface layer *h*_s_. This is a challenge faced in the interphase surface approach^29^. However, the surface layer thickness is always assumed to be 1 nm in the study of surface thermodynamic properties based on the facts: (1) both theoretical calculations and experimental observations focusing on the similar structure of grain-boundaries have been confirmed and widely accepted that their thickness variations are within the range of 0.4 to 2.0 nm^30^,^31^,^32^,^33^ and (2) according to the relaxation process of metallic films by MD simulations, the normal-relaxation always occurs in the first five atomic surface layers with the traction-free surfaces, whereas most of the atomic layers in the film maintain undeformed compared with the stress-free bulk crystal^34^. The thickness of the first five atomic surface layers is the double of the lattice constant, which is around 1 nm. Hereafter, we assume that the surface thickness is 1 nm to study the size-dependent electrochemical properties of the solid films.

### Size-dependent surface stress of the solid film

At the equilibrium, the biaxial surface stress of the solid film is given by


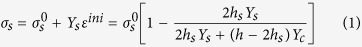


where *σ*_*s*_^*0*^ is the biaxial eigenstress in the surfaces, *ε*^*ini*^ is the initial strain after parallel relaxation, *h*_*s*_ and *h* are the thicknesses of the surface layer and the film, and *Y*_*s*_ and *Y*_*c*_ are biaxial Young’s modulus of the surface and the core, respectively.

**Figure 2:**
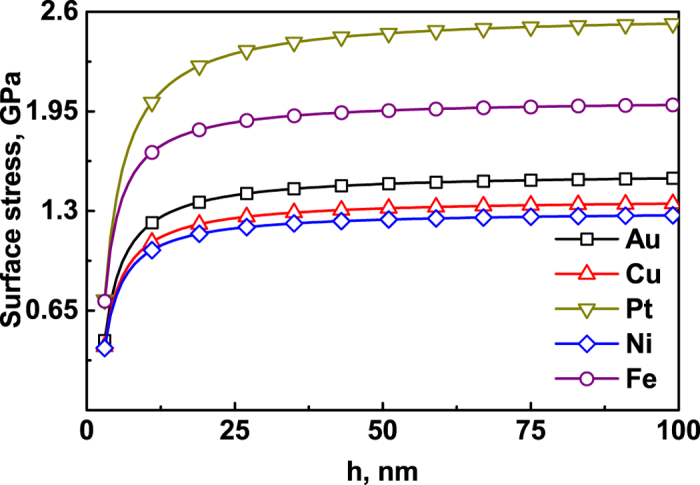
The film surface stress versus the thickness *h* with *h*_*s*_ = 1 nm, based on Eq. (1).

Substituting the data of *σ*_*s*_^*0*^, *Y*_*s*_, *Y*_*c*_ and *h*_*s*_ into Eq. (1), we can get the surface stress *σ*_*s*_ of the film versus the film thickness *h*, as shown in Fig. 2. In the smaller range of the film thickness, the absolute value of the surface stress increases sharply with increasing thickness *h*. For sufficiently thick films, the surface stress is almost the same as the surface eigenstress of the bulk material.

### Size-dependent equilibrium potential of the solid film

The equilibrium potential of the solid film can be described as:


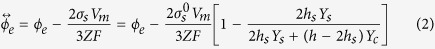


where *ϕ*_*e*_ is the equilibrium potential of the electrode reaction, *Z* is the number of transferred electrons, *F* is the Faraday constant, and *V*_*m*_ is the molar volume of the electrode material. When the thickness of the film *h* → ∞, that is, for the bulk materials, Eq. (2) can be given as:


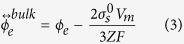


Eq. (3) is the general formulas of the equilibrium potential for bulk materials, which take the surface stress into account.

Based on Eq. (2) the equilibrium potential difference between the solid films and the bulk material can be described as the following:


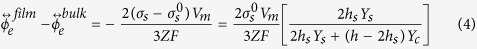


**Figure 3:**
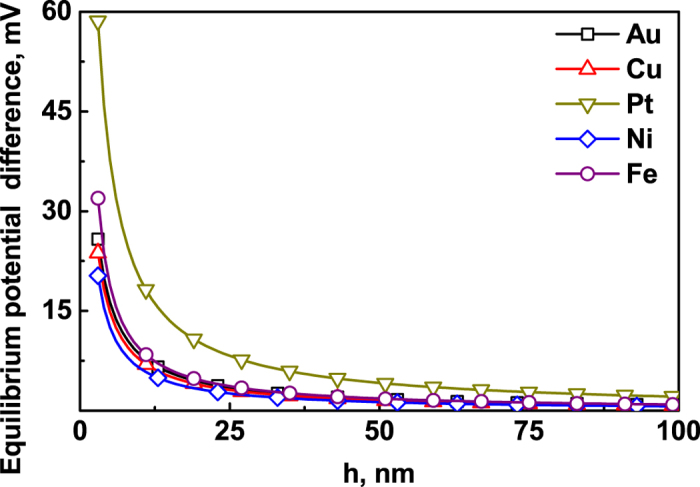
The equilibrium potential difference between the solid films and bulk material versus the film thickness *h* with *h*_*s*_ = 1 nm, based on Eq. (4).

Substituting the values in Table 1 into Eq. (4), the equilibrium potential difference between the films and the bulk material versus the film thickness can be received, as shown in Fig. 3. The value of the film equilibrium potential is higher than that of the bulk material. With increasing film thickness *h*, the difference between the solid films and the bulk material decreases to zero.

According to Eq. (4), one can obtain the standard electrode potential of the films *E*_*e*_^*film*^ corresponding to the reference electrode of the standard hydrogen electrode (SHE) compared with the bulk material value as a function of the film thickness *h*:


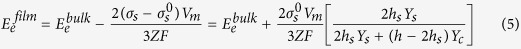


where *E*_*e*_^*bulk*^ is the standard electrode potential corresponding to the reference electrode SHE of the bulk material and listed in Table 1.

**Figure 4:**
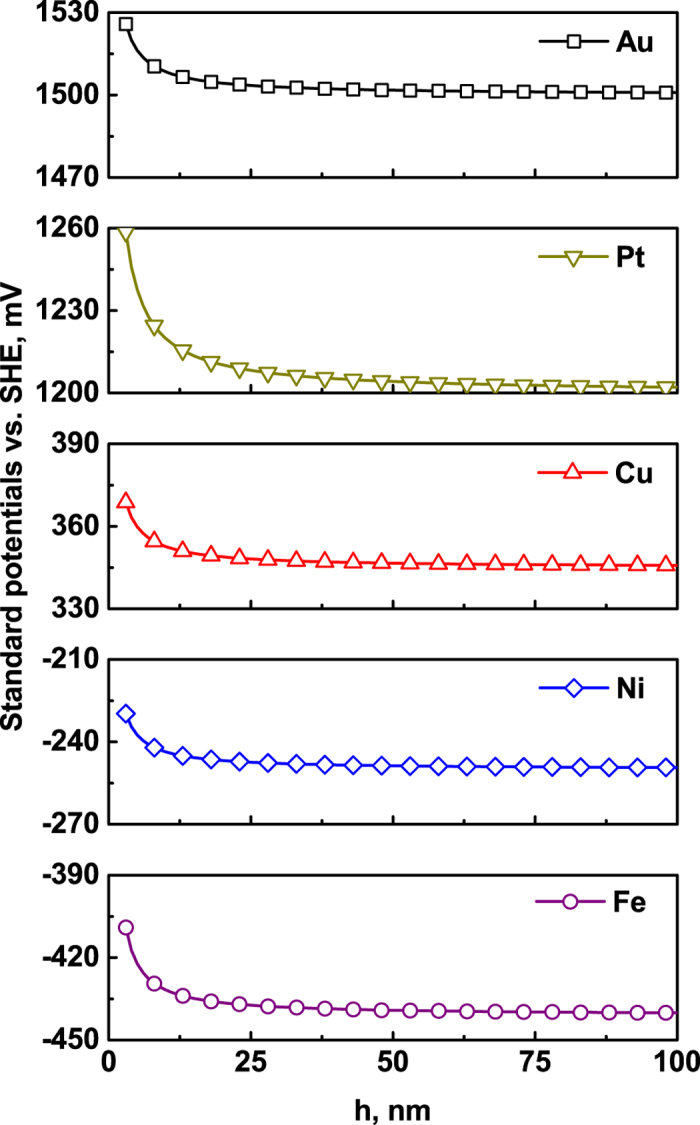
The standard potential of solid films, referred to the SHE, versus the film thickness *h*with *h*_*s*_ = 1 nm, based on Eq. (5).

Figure 4 shows the standard potential of solid films, referred to the SHE, versus the film thickness *h*. This is similar to the equilibrium potential difference between the film and the bulk material versus the film thickness. The value of the standard potential of the solid film is higher than that of the bulk material, and decreases with increasing thickness of the film.

### Size-dependent anodic current density

The surface stress existing in the film only modified the rate of the anodic reactions, i.e. the forward direction of the metal electrode reactions. The current density of the electrochemical reaction of the metal film electrode incorporate the effect of the surface stress can be described as:


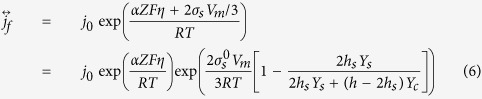



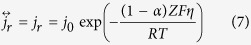



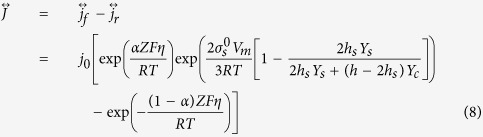


where *α* is the transfer coefficients of the electrochemical reaction, *η* is the overpotential, *R* is the gas constant, *T* is the absolute temperature, *j*_*0*_ is the exchange current density when the electrode reaction is in the equilibrium state (*η* = 0 and *j*_*f*_ = *j*_*r*_).

When the thickness of the film *h* → ∞, that is, for the bulk materials, Eq. (8) can be given as:


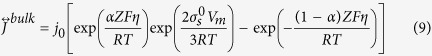


Eq. (9) is the general formulas of the electrochemical reaction rate for bulk materials, which take the surface stress into account.

Based on Eq. (6) one can get the current density ratio of the anodic reactions 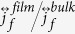 as a function of the film thickness *h*.


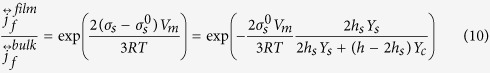


**Figure 5:**
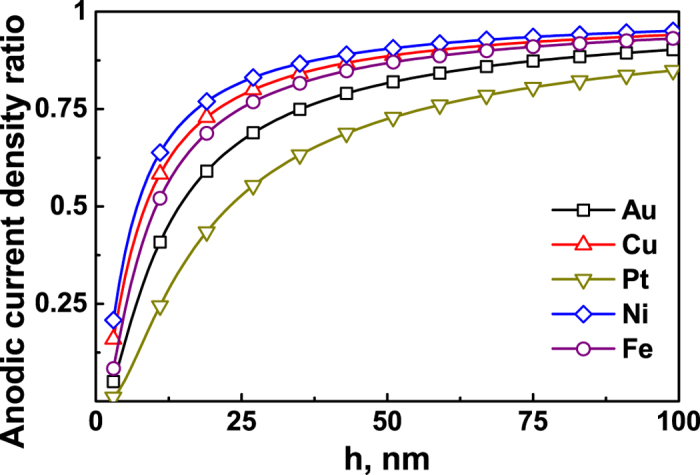
The current density ratio of the anodic reactions between solid films and bulk materials versus the film thickness *h* with *h*_*s*_ = 1 nm, based on Eq. (10).

By substituting the data in Table 1, the current density ratio of the anodic reactions between the films and the bulk material versus the film thickness can be received, as shown in Fig. 5. The oxidation current density of the free-standing metal film is lower than that of the bulk material, especially when the value of *h* is small. The difference will be close to zero when the *h* value is large enough.

## Discussion

The present study focuses on the fundamental elastic properties of a solid surface and considers solid films as typical structures to simplify the theoretical analysis. Usually, there are three common approaches to study the properties of surfaces^29^: the sharp or two-dimensional (2D) surface approach, the diffusive and/or interphase surface approach. Both the diffusive and interphase surface approaches treat surfaces as three-dimensional (3D). In the sharp surface approach, a single dividing interface of zero thickness^35^ is used to separate a studied system from its environment and the surface contribution to the thermodynamic properties is defined as the excess over the values that would obtain if the studied system and environment retained their properties constant up to the dividing interface. The diffusive interface is described by a gradient term, e.g., the concentration gradient^36^. The interphase approach treats an interface as a thermodynamic phase, and is usually chosen to be at locations where the properties are no longer varying significantly with the position. The interphase surface has a finite volume (thickness) and may be assigned thermodynamic properties in the normal way. Since the atoms within a very thin layer near surfaces experience a different local environment form that experienced by atoms in bulk, the physical properties and mechanical response of surfaces will be distinct from those of bulk materials. The surface modulus and the interphase interfaces have been generally used in the thermodynamic and mechanical researches of solid surfaces^29^,^37^. Therefore, the interphase approach is used in the present study to solve the problem of the size dependent electrochemical response of the solid films.

When a solid film is separated from the stress-free bulk substrate, the films will relax reaching equilibrium to meet the energy minimization requirements due to the creation of a new surface. The relaxation process can be separated into normal and parallel relaxation^34^. After normal relaxation, an eigenstress *σ*_*s*_^*0*^ is created in the surface layer, which is equal to the surface stress *σ*_*s*_^*bulk*^ of the bulk material. After parallel relaxation, a stress (or initial strain *ε*^*ini*^) will be generated in the core to balance the surface stress. The surface layers must undergo the same deformation as the core, because the surfaces are coherently adhered to the core, which changes the surface stress from *σ*_*s*_^*0*^ to *σ*_*s*_.

If the film state after normal relaxation is taken as the reference configuration, the total potential energy of a solid film is given by:


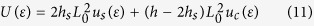


where *ε* denotes the biaxial strain, *u*_*s*_ and *u*_*c*_ are generalized energy densities per unit volume of the surface layer and the core, respectively, and *L*_0_ is the length and width of the film after normal relaxation. Further, *h*_*s*_ and *h* are the thicknesses of the surface layer and the film, respectively.

The energy density of the surface layer and the core can be written as:


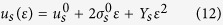



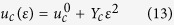


where *u*_*s*_^*0*^ and *u*_*c*_^*0*^ are the generalized energy densities of the surface layer and the core in the strain-free state, respectively.

Substituting Eqs (12) and (13) into Eq. (11), the total potential energy of a film is given by


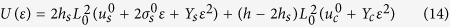


At the equilibrium, the energy minimization requires 
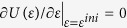
, which yields the generalized Young-Laplace equation^28^ to describe the mechanical force balance between the surface layer and the core:


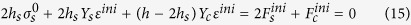


where 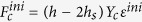 and 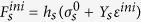 denote the core and the surface force per unit length, respectively.

From Eq. (15), the initial strain induced by the surface stress is given by


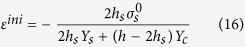


According to the Hooke’s law, the biaxial surface stress of the solid film is given by Eq. (1). From Eqs (16) and (1), it is clearly seen that the initial strain and the surface stress of the solid film is size-dependent, i.e. depend on the thickness of the film.

If we introduce 2D surface properties of the 2D surface eigenstress, 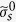, and 2D surface biaxial modulus, 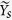, Eqs (15), (16) and (1) can be rewritten as


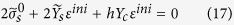



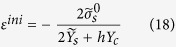



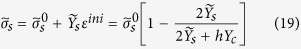


where 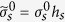 and 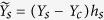. Eqs (17) and (18) are the Young-Laplace equation caused by the initial strain and the surface stress in the 2D surface approach^29^,^35^,^38^.

An elementary electrochemical reaction, developing on the metal electrode surface, can be written as:


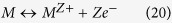


where *M* is the reduced state of a metal, *M*^*Z*+^ is the oxidized metal state, *e*^−^ is the electron charge, and *Z* is the number of transferred electrons. Here the anodic direction is considered to be forward and the cathodic direction is reversed. In the anodic direction the metal ions dissolved in the electrolyte from the electrode surface and a current generated from the electrode to the electrolyte.

In general, the electrochemical potential can be expressed as^25^,^26^


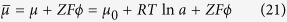


where *μ* is the chemical potential of the corresponding particles, *ϕ* is the inner electrical potential of the corresponding phase, *a* is the activity coefficient, *μ*_*0*_ is the standard chemical potential when *a* = 1.

In equilibrium, for Eq. (20), the Gibbs free energy Δ*G* = 0, thus


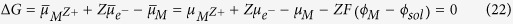


where *ϕ*_*M*_ and *ϕ*_*sol*_ are the inner electrical potential of the metal electrode and the electrolyte, respectively. Thus, the equilibrium potential of the electrode reaction can be written as:^25^,^26^


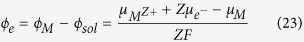


Then, for a given overpotential *η* the net electrochemical reaction rate can be obtained from the well-known Butler-Volmer equation^25^,^26^:


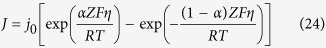


where 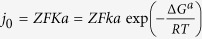 is the exchange current density when the electrode reaction is in the equilibrium potential *ϕ*_*e*_ (*η* = 0), *K* is the rate constant, *k* is the pre-exponential factor, and Δ*G*^*a*^ is the forward or reverse activation energy barrier.

It is well known that for solids there is a linear relationship between the Gibbs free energy and the pressure *P*. The volume *V* expanded into exponential series with respect to pressure is mainly described by the zero order term *V*_0_ because of the low compressibility of the solid phase. According to the Gibbs-Duhem equation^27^, Σ *N*_i_d*μ*_i_ = −*S*d*T* + *V*d*p*, the linear form of the chemical potential dependence on pressure follows the expression^12^:


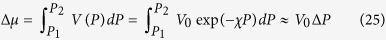


where *χ* is the compressibility coefficient of the solid (*χ* ≈ 10^−6^, *χP* ≪ 1).

If the metal electrode in a system of identical positive ions is simultaneously subjected to the effect of two external factors: mechanical and electrical, the mechano-electrochemical potential can be obtained by combining Eqs (21) and (25)^12^:


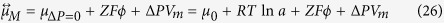


where *μ*_Δ*P*=0_ is the chemical potential when Δ*P* = 0 and *V*_m_ is the molar volume of the electrode material. The pressure Δ*P*, applied to the solid metal electrode, has no effect on the electrolyte phase.

Consequently, substituting Eq. (26) into Eq. (22), in the equilibrium state yields:


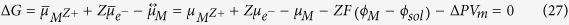


Then the equilibrium potential of the electrode reaction under the excess pressure Δ*P* can be written as^12^:


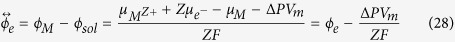


Thus, the change of the equilibrium potential due to the applied pressure is


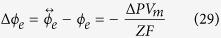


If the activity coefficient of the intermediate complex does not depend on the potential jump at the electrode-electrolyte boundary, the change of the potential barrier can be regarded as zero when counting the polarization potential Δ*ϕ*. Here, we have introduced the assumption that the potential barrier Δ*G*^*a*^ is independent from the Δ*ϕ* value^12^.

In the event of any mechanical deformation of the electrode, there would be a shift in the chemical/mechano-electrochemical potential of the metal electrode *M*, based on Eqs (25) and (26), denoted as Δ*PV*_*m*_. Correspondingly, the free energy of the activation barriers 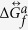 in the presence of a stress field is given as:


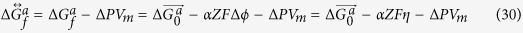


So,


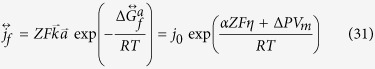



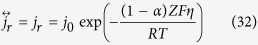


The current density of the electrode reactions in the presence of the stress are expressed as^12^:


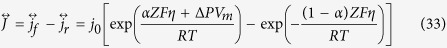


Because applying both the tension and compression stress can increase the chemical potential of the electrode materials, the symbol Δ*P* refers to the absolute value of the hydrostatic part of the stress tensor.

Surface stress is the key factor for the equilibrium potential and the reaction rate of the electrode reactions, which take place only on the electrode surface. From the biaxial surface stress (*σ*_*s*_) of the solid film, one can obtain the hydrostatic part of the stress tensor using Δ*P* = 2*σ*_*s*_/3. By substituting Δ*P* into Eq. (28), the equilibrium potential of a solid film can be described by Eq. (2).

By substituting Δ*P* into Eqs (31, 32, 33), the current density of the electrochemical reaction of the solid film can be described by Eqs (6, 7, 8).

## Methods

### MD simulations

MD simulations were conducted to study the intrinsic surface elastic parameters of solid films^38^. MD simulations for Au, Pt, Ni, Cu face-centered-cubic crystals and a Fe body-centered-cubic crystal were performed with the LAMMPS code^39^. All crystals were simulated with the embedded-atom method potentials^40^,^41^. All simulations were conducted in a molecular statics framework and implemented by using the conjugate gradient method.

#### Bulk biaxial Young’s modulus

The simulations were performed on bulk crystals to obtain the bulk biaxial Young’s modulus. A representative domain of 8 × 8 × 8 unit cells with periodic boundary conditions (PBCs) in all three directions was adopted to simulate the bulk material. The reference energy *U*_0_ and equilibrium lattice constant in the stress-free bulk crystals were obtained through energy minimization. The bulk biaxial modulus was then determined from simulations of biaxial compressive and tensile tests^38^, which were conducted in two steps. (1) All atoms were displaced uniformly in the *xy* plane according to the uniform biaxial strain with an increment of 0.1%. (2) The plane stress condition in the *z* direction was identified by adjusting the periodic length along the *z* direction to achieve the minimum total potential energy configuration. A strain range of −1% to 1% was adopted, corresponding to the initial strains in thin films, discussed in the next section.

**Figure 6:**
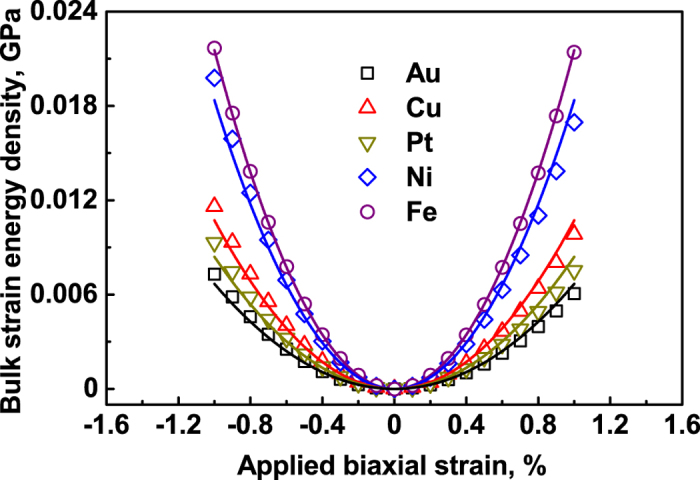
Strain energy per unit volume as a function of the biaxial strain, where solid lines are the fitting curves using (*U*_c_ − *U*_0_) = *Y*_c_ *ε*_c_^2^ and biaxial strain loading is applied along the [100] and [010] directions, while the [001] direction is traction free.

Since the unloading data overlap completely with the loading data indicates that the bulk crystals deform elastically within the applied strain range. Furthermore, under the traction-free conditions along the *z* direction, the strain energy density (*U*_c_ − *U*_0_) versus the applied biaxial strain *ε*_c_ was well fitted using a quadratic function (*U*_c_ − *U*_0_) = *Y*_c_ *ε*_c_^2^, as shown by the solid lines in Fig. 6, where *Y*_c_ is the bulk biaxial Young’s modulus was calculated from the second derivatives of the strain energy with respect to the applied strain. These bulk biaxial Young’s modulus *Y*_c_ were listed in Table 1.

#### Surface eigenstress and surface biaxial Young’s modulus

For solid film simulations, a film was created by placing atoms with the stress-free bulk lattice constant. To simulate a representative element of an infinitely large film, PBCs were applied only in the *x* and *y* directions with the free (001) surfaces in the *z* direction. Film thickness *h* was determined from the volume^38^ of the simulated representative film in its undistorted configuration with the film length and width equal *L*_0_. The undistorted volume multiplied by the density of the bulk crystal represented the total mass of the atoms in the film. The film thickness ranged from 2 to 100 nm and the representative element had a size of 8 × 8 unit cells in the *x* and *y* directions. The relaxation of the solid film towards the minimal energy state was separated into two steps, i.e., normal and parallel relaxations^38^. In normal relaxation, atoms were allowed to move in the *z* direction to minimize the total energy with the prescribed representative film length *L*_0_ in both *x* and *y* directions. After normal relaxation, parallel relaxation was conducted, in which atoms were allowed to move in all three directions. For a given number of unit cells in the representative film, the total film energy depends on the representative film length *L*. When the parallel relaxation reaches the final equilibrium state, the energy is minimized and the representative film length has its initial value *L*^ini^. Once the initial representative film length was determined, the initial strain *ε*^*ini*^ of the film was calculated as *ε*^*ini*^ = ln(*L*^ini^/*L*_*0*_).

**Figure 7:**
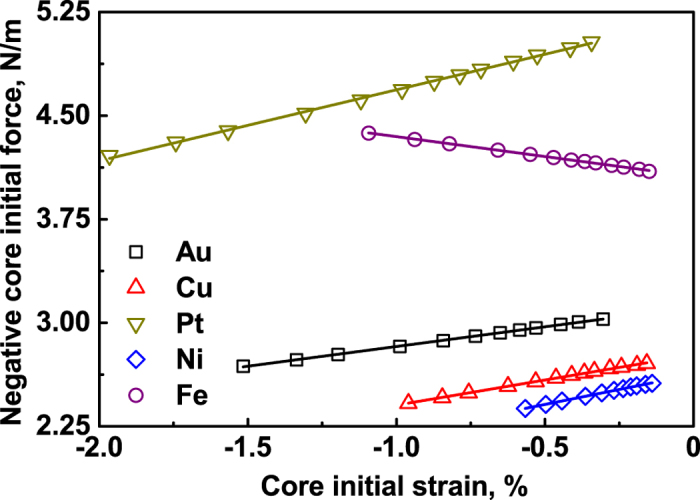
Negative core initial force plotted versus initial strain, for the (001) solid films, where solid lines are fitting curves using Eq. (17).

Then the fitting lines −*F*_*c*_^*ini*^ = −*hY*_c_ *ε*^ini^ plotted versus *ε*^ini^ yield the 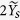 slope and the 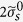 intersection, meaning that the 2D surface biaxial Young’s modulus 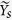 and eigenstress 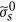 can be determined. Figure 7 shows the negative initial core force (−*F*_*c*_^*ini*^) as a function of the core initial strain (*ε*^ini^), where the solid lines are fitting results based on equation 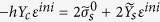.

The intrinsic surface elastic parameters of the eigenstress (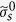) and the biaxial Young’s modulus (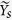), and the bulk biaxial Young’s modulus (*Y*_c_) of Pt, Au, Ni, Cu and Fe are summarized in Table 1.

### Theoretical analysis

Thermodynamic expressions for the size-dependent surface stress, equilibrium potential, and the anodic current density in the solid films were developed based on the MD simulations. Furthermore, an extended form of the popular Butler-Volmer equation was used for modeling electrode reaction rates. The shift in the chemical potential used in the Butler-Volmer equation was determined using the Gibbs-Duhem equation. The theoretical analysis and methodology developed in the present work shows that the surface stress, equilibrium potential, and the oxidation current density in the solid films were size-dependent. The anodic current density of a metal film electrode reaction increased and the equilibrium potential decreased with increasing solid film thickness due to the surface energy/stress of the solid films decreasing with respect to bulk materials.

## Additional Information

**How to cite this article**: Ma, H. *et al*. Eigenstress model for electrochemistry of solid surfaces. *Sci. Rep.* **6**, 26897; doi: 10.1038/srep26897 (2016).

## Figures and Tables

**Table 1:** Eigenstress 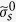 and *σ*_*s*_^*0*^, surface modulus 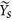 and *Y*_s_, bulk modulus *Y*_c_, number of electronic charge *Z* and the standard electrode potential *E*_*e*_^*bulk*^ referred to the SHE.

Materials	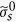 , N/m	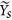 , N/m	*σ*_*s*_^*0*^, GPa	*Y*_s_, GPa	*Y*_c_, GPa	*Z*	*E*_*e*_^*bulk*^, V
Pt (001)	2.60	25.73	2.60	109.80	84.08	2	1.20
Au (001)	1.56	14.27	1.56	81.07	66.81	3	1.50
Ni (001)	1.31	21.67	1.31	205.32	183.65	2	−0.25
Cu (001)	1.38	18.20	1.38	125.43	107.23	2	0.35
Fe (001)	2.03	−14.57	2.03	200.92	215.48	2	−0.44

(*σ*_*s*_^*0*^ and *Y*_s_ were calculated based on *h*_s_ = 1 nm and the surface of (001)).
